# Vaccine Uptake and Intentions: Insights from a Texas Survey on Factors Influencing COVID-19 Vaccination Decisions

**DOI:** 10.3390/vaccines12060601

**Published:** 2024-05-31

**Authors:** Yordanos M. Tiruneh, Paula M. Cuccaro, Kimberly S. Elliott, Jing Xie, Journey Martinez, Mark Owens, Christian R. Alvarado, Jose-Miguel Yamal

**Affiliations:** 1 Department of Preventive Medicine and Population Health, School of Medicine, University of Texas at Tyler, Tyler, TX 75708, USA; 2 Department of Internal Medicine, School of Medicine, University of Texas at Tyler, Tyler, TX 75708, USA; 3 Department of Internal Medicine, Division of Infectious Diseases, University of Texas Southwestern Medical Center, Dallas, TX 75390, USA; 4 Center for Health Promotion and Prevention Research, Department of Health Promotion and Behavioral Sciences, School of Public Health, University of Texas Health Science Center at Houston, Houston, TX 78701, USA; paula.m.cuccaro@uth.tmc.edu; 5 Department of Health Policy, Economics, and Management, School of Professions, University of Texas at Tyler, Tyler, TX 75708, USA; kimberly.elliott@uthct.edu; 6 Coordinating Center for Clinical Trials, Department of Biostatistics and Data Science, School of Public Health, University of Texas Health Science Center at Houston, Houston, TX 77030, USA; jing.xie@uth.tmc.edu (J.X.); journey.martinez@uth.tmc.edu (J.M.); jose-miguel.yamal@uth.tmc.edu (J.-M.Y.); 7 Department of Political Science, School of Humanities and Social Sciences, The Citadel, Charleston, SC 29409, USA; mowens6@citadel.edu; 8 Department of Epidemiology and Biostatistics, School of Medicine, University of Texas at Tyler, Tyler, TX 75708, USA; christian.alvarado@uttyler.edu

**Keywords:** COVID-19, vaccine hesitancy, vaccine acceptance, vaccine uptake, vaccine intentions, barriers, Texas

## Abstract

The effectiveness of COVID-19 vaccines depends on widespread vaccine uptake. Employing a telephone-administered weighted survey with 19,502 participants, we examined the determinants of COVID-19 vaccine acceptance among adults in Texas. We used multiple regression analysis with LASSO-selected variables to identify factors associated with COVID-19 vaccine uptake and intentions to receive the vaccine among the unvaccinated. The prevalence of unvaccinated individuals (22%) was higher among those aged 18–39, males, White respondents, English speakers, uninsured individuals, those facing financial challenges, and individuals expressing no concern about contracting the illness. In a fully adjusted regression model, higher odds of being unvaccinated were observed among males (aOR 1.11), the uninsured (aOR 1.38), smokers (aOR 1.56), and those facing financial struggles (aOR 1.62). Conversely, Asians, Blacks, and Hispanics were less likely to be unvaccinated compared to Whites. Among the unvaccinated, factors associated with stronger intent to receive the vaccine included age (over 65 years), Black and Hispanic ethnicity, and perceived risk of infection. Hispanic individuals, the uninsured, those covered by public insurance, and those facing financial challenges were more likely to encounter barriers to vaccine receipt. These findings underscore the importance of devising tailored strategies, emphasizing nuanced approaches that account for demographic, socioeconomic, and attitudinal factors in vaccine distribution and public health interventions.

## 1. Introduction

The effectiveness of COVID-19 vaccines in reducing the impact of the pandemic depends largely on widespread uptake. According to the Centers for Disease Control and Prevention (CDC), COVID-19 vaccine uptake rates remain lower than expected. As of December 2023, 18.5% of adults in the United States had not received the vaccine [1]. Furthermore, national surveys have highlighted geographical disparities in vaccine uptake, with southern states, including Texas, falling behind in vaccination rates. In 2003, Texas had an adult unvaccinated rate of 24%, which is higher than the national average [1]. Understanding the contextual factors that shape vaccine decisions among these populations is crucial to developing effective public health strategies [2].

While vaccine hesitancy is not a new concept, exploring COVID-19 vaccine acceptance across demographic groups can lead to tailored and targeted strategies [3]. Since COVID-19 is likely to exhibit a lasting and seasonal resurgence, public health officials must ensure that COVID-19 vaccines are accessible and that clear information regarding their effectiveness is readily available. COVID-19 vaccine decisions differ conceptually from those involving vaccines for other diseases with well-established availability due to multifaceted and evolving factors. Additionally, complex historical trends related to race and ethnicity, including medical mistrust and experiences of discrimination among minority communities, further complicate the vaccine-acceptance discourse [4,5,6].

Various factors, including sex, race, age, education, and income, influence an individual’s decision regarding vaccine acceptance. Concerns over vaccine safety, including development timelines and potentially unpleasant side effects, as well as beliefs and ideologies, perceived infection risk, individual susceptibility, and medical mistrust, all contribute to COVID vaccine acceptance decisions [7,8,9,10,11,12]. Research indicates that vaccine hesitancy varies across different population groups and locations, evolving over time along a continuum [13]. Evaluating contextual factors associated with vaccination decisions is necessary to gain insights that can inform potential interventions aimed at enhancing vaccination uptake or addressing barriers [14]. 

The COVID-19 pandemic has exacerbated pre-existing disparities based on race, ethnicity, and socioeconomic disadvantages. Observed discrepancies in vaccine uptake among minority or underserved population groups further widen existing disparities in COVID-19 infection rates and outcomes [15]. Previous studies on vaccine uptake in Texas focused on particular demographics, such as border communities [16] and various sub-regions [17,18,19], as well as among health professionals [18,20,21]. While these studies have shed light on vaccine hesitancy, an approach encompassing a wider spectrum of age groups and geographic regions would offer a more comprehensive understanding of vaccine decisions across the entire state. This study seeks to delve into the underlying factors shaping vaccination decisions among residents of Texas. This study aims to (1) examine predictors of adult vaccine acceptance in Texas following the full rollout of the vaccine for multiple age groups, including booster doses; (2) assess vaccine intentions among unvaccinated individuals; and (3) identify factors associated with barriers to accessing the COVID-19 vaccine.

## 2. Materials and Methods

The data used for this cross-sectional study were collected from responses to the COVID-19 Vaccine Hesitancy and Confidence (COVHAC) Survey: A Rapid Community Assessment, which was conducted from 4 April 2022 to 14 November 2022. Data collection began shortly after masks were no longer mandated for airline travel. The survey was concluded a month after the CDC and FDA approved the COVID-19 vaccine for infants, toddlers, and preschool children. During the data-collection period, reported infections increased from 2,367,077 (as of 2 April 2022) to 6,401,754 (as of 15 November 2022) [22], and the number of vaccinations that had been administered in Texas increased from 45,390,689 prior to the data-collection period to 50,672,487 (as of 16 November 2022) [23]. 

### 2.1. Study Design and Setting

The COVHAC Survey was a telephone-administered survey conducted statewide among adults over the age of 18 years. The survey items were collaboratively developed by the funder and the researchers involved in the study. The survey collected demographic information, the respondent’s health status (including vaccination status), barriers and intentions regarding vaccination, and factors hindering or facilitating vaccine uptake. This study was approved by the UT Tyler Institutional Review Board and the UTHealth Houston Committee for the Protection of Human Subjects prior to study initiation. Verbal informed consent was obtained at the beginning of each call.

The survey used address-based sampling to identify homes with a cell phone or a landline. Approximately 85% of the phone numbers were cell phones, and 15% were landlines. Eligible participants were selected randomly using Texas area code phone numbers and included both English and Spanish speakers. Survey calls were made from three call centers in Texas between 9 a.m. and 9 p.m., primarily made during the evening and weekends, with limited hours of daytime calling. Three phone calls were made to each phone number to limit any bias due to the availability of a potential respondent. One call center (call center 3) used targeted oversampling of census-block groups with higher reported rates of unvaccinated children at home (collected 27 June 2022–1 August 2022). This set of responses did not significantly increase the number of responses from parents of unvaccinated children, and the decision was made to cease targeting and return to random sampling only for all call centers. Interviewers read the questions using Computer-Assisted Telephone Interviewing (CATI) software (Voxco Version 6.5.21228.10055). 

**Figure 1:**
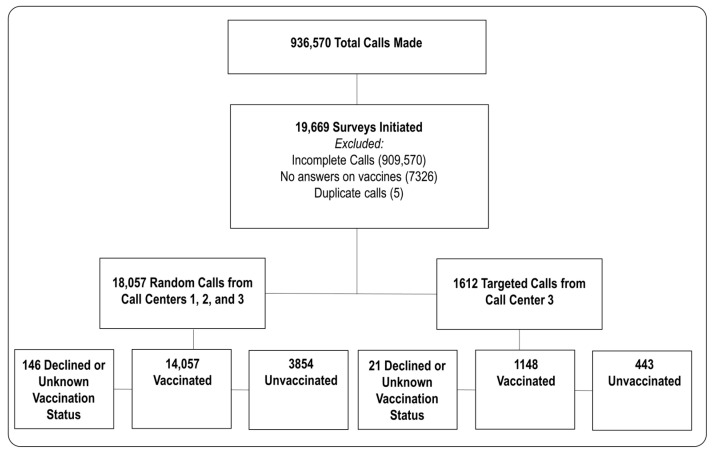
Consort Flow Diagram of Participant Recruitment.

The sample size was established considering feasibility, practical constraints, and mutually agreed-upon objectives between the funder and the investigators. A total of 936,570 calls were made, and 19,669 individuals initiated the survey. The response rate of the survey was 2.1%, and this analysis was restricted to 19,502 respondents who answered at least one vaccination question (Figure 1). The survey script prominently emphasized that participation was voluntary, granting respondents the autonomy to decline to answer any question or discontinue the survey at any point. No form of gift or monetary compensation was provided in exchange for participating in our survey.

### 2.2. Survey Weights

To generalize the findings to the Texas population (based on 2021 American Community Survey 5-year population estimates) and to account for the targeted sampling design, survey weights were calculated for age group, sex, race/ethnicity, and public health region (PHR), with the base weight being the product of each individual weight. These weights corresponded to the number of people in the population that each individual represented, calculated as the ratio of No. in the subgroup in the population to No. in the subgroup in the survey. Subsequently, the final survey weight was standardized by dividing each base weight by the average of the base weights.

### 2.3. Outcomes

Our primary outcome of interest was self-reported vaccination status, determined by whether a respondent reported having received at least one dose of any COVID-19 vaccine. Secondary outcomes included whether an unvaccinated respondent expressed the intent to receive a COVID-19 vaccine within the subsequent three months following the survey, along with the identification of barriers experienced among unvaccinated individuals.

### 2.4. Covariates

Covariates included respondent demographics and health-status information, including geographic region within Texas (public health region [24]), age group, sex (categorized as male, female, or other), race/ethnicity (grouped as Hispanic, non-Hispanic—White, Asian, Black, Multiracial, and Other), language (English, Spanish, Other), employment status (working, not working), reason for not working (if applicable), and whether the reason for not working was COVID-related (yes/no). The state of Texas comprises eleven Public Health Regions (PHRs), each overseen by corresponding public health offices. These offices are located in Lubbock (Region 1), Arlington (Regions 2/3), Tyler (Regions 4/5), Houston (Region 6/5), Temple (Region 7), San Antonio (Region 8), El Paso (Regions 9/10), and Harlingen (Region 11). The regions are delineated to ensure that each has a manageable population size and similar demographic characteristics, which facilitates more effective public health management and service delivery through tailored interventions and resource allocation to address specific health needs within each region. Adverse health outcomes exhibit varying severity across these different PHRs. Insurance status was recategorized as private, public, uninsured, or other. Comorbidities such as cancer, COPD, heart disease, obesity, sickle cell disease, diabetes, immunocompromised conditions, tuberculosis, HIV, hypertension, and other chronic diseases were also recorded. Additional data included smoking status, COVID-19 infection history, and whether individuals encountered difficulties in accessing basic necessities during the pandemic. Respondents were also asked about their personal concerns regarding illness, dichotomized as “somewhat or extremely concerned” versus “otherwise,” including their concerns about illness in others.

### 2.5. Statistical Analysis

Chi-square tests were used to evaluate differences in baseline characteristics by vaccination status. All tests were two-sided. Primary and secondary outcomes were analyzed using multiple logistic regression models adjusted for each included covariate. Covariates were selected using LASSO regression, where lambda was selected using ten-fold cross-validation. Standardized survey weights were utilized in both LASSO variable selection and in assessing model fit for the outcomes regarding whether a respondent was vaccinated (primary outcome) and whether unvaccinated individuals expressed the intention to receive the vaccine (secondary outcome). No weighting was used for the secondary outcome of an indication that a respondent intended to be vaccinated. We omitted the “Other” category from the sex variable because we lacked a population denominator for the application of survey weights. Generalized variance inflation factors were examined to rule out multicollinearity. The goodness of fit of the model was assessed using the Hosmer-Lemeshow test and the area under the receiver operating characteristic curve. Model diagnostics, including exploration of the effects of removing potentially influential data points, were assessed. No significant deviations were identified. A result returning a *p*-value < 0.05 was deemed statistically significant. All analyses were conducted using R version 4.3.0 (R Foundation for Statistical Computing).

## 3. Results

Of the 19,502 individuals who responded to the question regarding vaccination status, 4297 (22%) reported being unvaccinated (Table 1). Most respondents resided in PHR 2/3 or 6/5S and were 50 years of age or older (53.7%). Women respondents (51.2%) slightly outnumbered men, and the majority identified as White (50.7%), spoke English at home (86.1%), worked in-person (59.2%), and were covered by private insurance (55.8%). A higher proportion of younger individuals aged 18–39 years (38.5% vs. 26.6%; *p* < 0.001), males (54.1% vs. 45.5%; *p* < 0.001), White individuals (57.2% vs. 48.9%; *p* < 0.001), and those who spoke English at home (90.1% vs. 85.0%; *p* < 0.001) reported being unvaccinated (vs. vaccinated). Additionally, a greater percentage of individuals residing in PHR 4/5N 11.9% vs. 6.8%; *p* < 0.001), the uninsured (16.1% vs. 8.7%; *p* < 0.001), and those who were employed (70.6% vs. 61.3%; *p* < 0.001) were more likely to be unvaccinated. Among those employed, individuals working in-person (64.9% vs. 57.3%; *p* < 0.001) and those experiencing financial difficulties during the pandemic (25.9% vs. 19.3%; *p* < 0.001) were more likely to be unvaccinated. Additionally, current smokers were more likely to be unvaccinated (12.4% vs. 7.8%; *p* < 0.001).

In contrast, a higher proportion of individuals reported suffering from cancer (5.0% vs. 3.3%; *p* < 0.001), diabetes (13.8% vs. 7.7%; *p* < 0.001), hypertension (19.3% vs. 10.2%; *p* < 0.001), heart disease (8.0% vs. 5.5%; *p* < 0.001), obesity (14.4% vs. 10.0%; *p* < 0.001), and chronic conditions (5.4% vs. 3.6%; *p* < 0.001) were vaccinated. Moreover, significantly greater proportions of individuals reporting no concern about illness for themselves (72.3% vs. 33.4%; *p* < 0.001) or others (55.2% vs. 25.6%; *p* < 0.001) were unvaccinated. Similarly, lower proportions of individuals with a history of COVID-19 infection that required medical care (20.8% vs. 23.6%; *p* < 0.001) or a COVID-19 infection that did not need medical care (23.7% vs. 34.6%; *p* < 0.001) were vaccinated.

**Figure 2:**
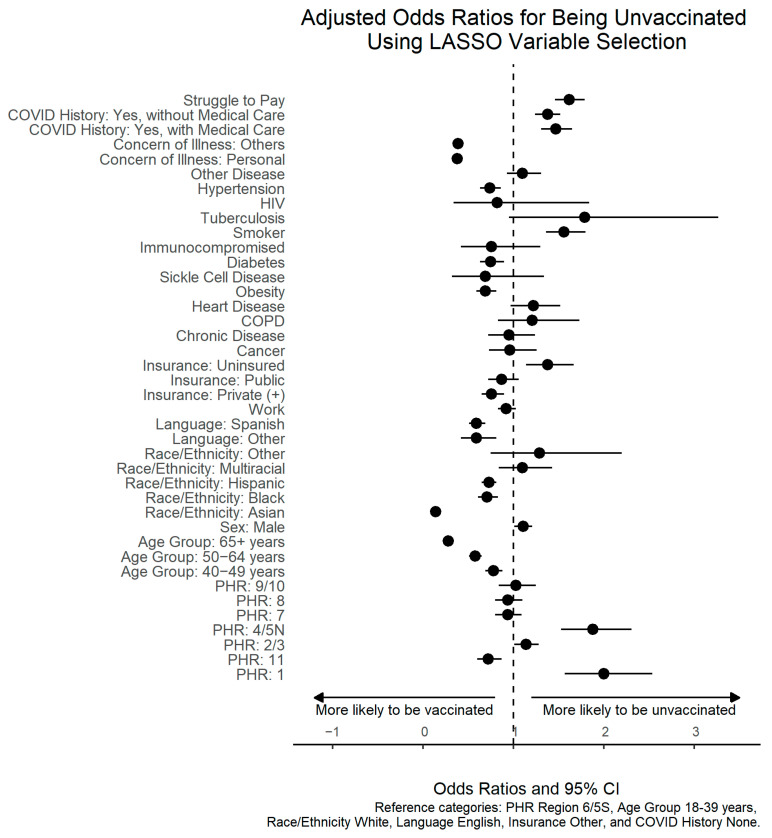
Adjusted Odds Ratio for Being Unvaccinated Using LASSO Variable Selection. Odds ratio and 95% CI. Reference categories: PHR Region: 6/5S, Age Group: 16–39 years, Race/Ethnicity: White, Language: English, Insurance: Other, COVID History: None.

Our fully adjusted model with LASSO-selected variables identified multiple factors associated with being unvaccinated (Table 2; Figure 2). Except for PHRs 11, 7, and 8, all other regions exhibited higher odds of being unvaccinated compared to PHR 6/5S. Compared with younger people (18–39 years), older individuals who were 40–49 years of age (aOR, 95% CI: 0.78, 0.69–0.88), 50–64 years of age (aOR, 95% CI: 0.58, 0.51–0.65), and over 65 years of age (aOR, 95% CI: 0.28, 0.23–0.34) had lower odds of being unvaccinated. Males (aOR, 95% CI: 1.11, 1.01–1.21), the uninsured (aOR, 95% CI: 1.38, 1.14–1.67), smokers (aOR, 95% CI: 1.56, 1.36–1.80), and individuals facing financial difficulties during the pandemic (aOR, 95% CI: 1.62, 1.46–1.79) were more likely to be unvaccinated than their counterparts (Table 2). In contrast, there was a lower likelihood of being unvaccinated among individuals identifying as Asian (aOR, 95% CI: 0.14, 0.10–0.19), Black (aOR, 95% CI: 0.71, 0.61–0.83), and Hispanic (aOR, 95% CI: 0.73, 0.65–0.81) compared to White respondents. Participants who speak Spanish (aOR, 95% CI: 0.59, 0.51–0.69) or other languages at home (aOR, 95% CI: 0.59, 0.42–0.81) had lower odds of being unvaccinated compared to English-speaking participants. Similarly, statistically significant lower odds of being unvaccinated were observed among individuals with obesity (aOR, 95% CI: 0.69, 0.59–0.81), hypertension (aOR, 95% CI: 0.74, 0.63–0.86), and diabetes (aOR, 95% CI: 0.75, 0.63–0.90). Those who expressed concerns about themselves (aOR, 95% CI: 0.38, 0.34–0.42) or others becoming infected with COVID-19 were less likely to be unvaccinated than were those reporting no such concerns (aOR, 95% CI: 0.39, 0.35–0.43). Other factors associated independently with a higher likelihood of being unvaccinated are reported in Table 2.

Table 3 presents the results of adjusted analyses indicating the intention on the part of unvaccinated individuals to receive the COVID-19 vaccine within 3 months following the completion of the survey. Individuals over the age of 65 years were more likely than those between 18 and 39 years of age (aOR, 95% CI: 1.06, 0.60–1.85) to intend to be vaccinated. Black (aOR, 95% CI: 2.04, 1.14–3.55) and Hispanic individuals (aOR, 95% CI: 2.10, 1.33–3.29) had higher odds of harboring vaccine intentions than White participants. Likewise, Spanish-speaking individuals (aOR, 95% CI: 2.73, 1.67–4.46) or speakers of other languages (aOR, 95% CI: 1.47, 0.40–4.23) at home were more likely to receive the vaccine compared to English speakers. Individuals with concerns about illness for themselves (aOR, 95% CI: 3.35, 2.31–4.87) or others (aOR, 95% CI: 1.98, 1.37–2.88) were more likely to harbor positive vaccine intentions compared to those with no concerns. Conversely, those who were employed (aOR, 95% CI: 0.59, 0.40–0.85) were less likely to receive the vaccine than their counterparts (Table 3).

Among those with unknown or unvaccinated status, a greater proportion of Hispanic individuals reported facing barriers (vs. no barriers) to receiving the vaccine (35.2% vs. 24.6%; *p* = 0.001), as did those who speak Spanish (18.0% vs. 7.6%; *p* < 0.001) or other languages at home (5.4% vs. 2.0%; *p* < 0.001). Similarly, uninsured individuals (22.3% vs. 15.8%; *p* = 0.001), individuals covered by public insurance (34.0% vs. 21.6%; *p* < 0.001), and those who experience financial struggles (48.2% vs. 25.1%; *p* < 0.001) were more likely to experience barriers to receiving the vaccine. In contrast, a higher proportion of employed individuals (71.0% vs. 57.3%, *p* < 0.05) were less likely to experience barriers to accessing the vaccine. Individuals with extreme concerns about illness for themselves (19.1% vs. 3.2%, *p* < 0.001) or others (22.9% vs. 9.0%, *p* < 0.001) experienced more formidable barriers in accessing the COVID-19 vaccines. Several illnesses were independently associated with the likelihood of experiencing barriers to receiving the vaccine among unvaccinated individuals, as reported in Table 4.

## 4. Discussion 

We sought to explore factors associated with COVID-19 vaccination decisions using a statewide survey of a representative sample of 19,502 adults in Texas between April and November 2022. Our study revealed four pertinent findings. First, we observed lower rates of vaccine uptake in rural, underserved regions. Second, our analysis shows that females and ethnic minorities were more likely to be vaccinated against COVID-19 than their male and White counterparts. Third, a greater proportion of employed individuals, especially those who were working in-person during the pandemic, were more likely to be unvaccinated despite facing less formidable barriers to vaccine access. Fourth, uninsured individuals and those who were financially insecure during the pandemic were more likely than others to remain unvaccinated. Our study highlights several other sociodemographic, economic, health-related, and attitudinal factors associated with COVID-19 vaccine decisions. 

The rate of unvaccinated individuals in our sample was 22%, which closely aligns with the CDC’s estimated rate of 24% for Texas. This, along with the racial, geographic, age, and sex distributions, suggests that our sample was fairly representative. Furthermore, we observed disparities in COVID vaccine uptake across geographic regions, whereby individuals residing in PHR 1 and 4/5 North, largely rural and mostly under-resourced counties, were more likely to be unvaccinated compared to those living in PHR 6/5 South, which is a large, metropolitan region. Prior research has indicated that vaccine coverage tends to be lower in zip codes with the highest overall social vulnerability indices (SVI) [25], which could explain the observed disparities in our study. Additionally, political ideology [12,26,27,28,29], social vulnerability [25,30], rurality [27,31,32], and experienced or perceived racial discrimination in healthcare and medical services [30,33] shape vaccine uptake. Yet, residents in these under-vaccinated regions, especially those in PHR 1, expressed firmer intentions to receive the vaccine in the future, which is encouraging. Further studies in regions that exhibit disproportionately lower vaccine rates are warranted to understand the reasons for vaccine hesitancy and assess potential barriers to receiving the vaccine. 

In our sample, males were more likely to be unvaccinated than their female counterparts. Our data mirror the ImmTrac2 data on vaccinations from May 2023, where the vaccination rates in Texas (involving at least one vaccine) were 72% for males and 78% for females [34]. Other studies, however, including a prior study on vaccine intentions in Central Texas [13], as well as several other studies [35,36], present divergent results. A review and meta-analysis [37] of 60 studies also provided evidence that men are more likely to be vaccinated or have a firmer intention to receive the vaccine. We found that racial and ethnic minorities, specifically Black, Asian, and Hispanic individuals, were more likely to be vaccinated compared to White individuals. This result differs somewhat from the ImmTrac 2 data, which suggest that Blacks, Whites, and Hispanics exhibit the lowest rates of having had at least one vaccine. Asians have the highest vaccination rates [34]. Black and Hispanic participants also expressed firmer intentions of receiving the vaccine in subsequent months compared to White participants in our survey. This trend highlights an important demographic shift in vaccination acceptance in our sample, possibly influenced by the timing of our survey, which was conducted several months after the initial vaccine rollout. This observation could also suggest a transition from vaccine hesitancy to a “wait-and-see” approach, whereby individuals who initially harbored hesitations were waiting to observe potential health-related implications or side effects of the vaccine. Individuals who were not vaccinated but expressed concern still show interest in being vaccinated. This finding reinforces the importance of context; current concerns significantly influence a respondent’s plan to take action in the future. Public health officials could leverage their platforms to provide information, including personal testimonials from vaccinated individuals as proof of well-being and of a lower risk of suffering adverse health outcomes following vaccination. 

A higher proportion of employed individuals, especially those working in-person during the pandemic, remained unvaccinated, although work was not associated with being unvaccinated in our adjusted model. Individuals working in-person were more likely to be younger and healthier, which may have given them a false sense of reassurance regarding their risk and severity of infection [38]. Misconceptions about perceived risks could be internalized if the message came from apparently authoritative sources, such as employers. Moreover, for some people, working in-person was required to earn income during the pandemic, and such individuals are likely to trust information that supports their ability to continue working. This explanation is logical considering our finding that unvaccinated working adults showed lesser intent to be vaccinated in the future despite facing lower barriers to accessing the vaccine compared to their unemployed counterparts. The observed reluctance to become vaccinated among working adults could be attributed to concerns about the financial impact of vaccination if respondents would have to miss work time. Despite an extensive outreach effort and the availability of flexible hours for vaccination purposes, concerns about side effects and the potential risk of being unable to work without time off may be a reasonable financial disincentive for working individuals to be vaccinated. A study by Rosado et al. highlights a paradoxical trade-off in a demanding work environment, where essential workers were more likely to become vaccinated if a co-worker was sick than if a family member was sick [39]. 

Insurance status was strongly associated with vaccine status in our sample, with uninsured individuals being more likely to be unvaccinated. Those lacking insurance or covered by public insurance also encountered greater barriers to accessing the COVID-19 vaccine. This finding aligns with findings by other researchers, which demonstrated that individuals without health insurance were less likely to be vaccinated [40,41,42]. The underlying disparity could be attributed to uninsured individuals lacking primary care providers, who, therefore, enjoy fewer opportunities, are exposed to fewer reminders, and have limited convenient options for receiving the vaccine. Lo et al.’s research indicated that counties with a higher number of primary care providers (PCP) per 100,000 population had higher COVID-19 vaccination rates [43], highlighting the crucial role of primary care providers as a trusted source of information in positively impacting vaccine acceptance [44,45]. Additionally, despite the COVID-19 vaccine being available at no cost, concerns about associated expenses may deter uninsured individuals from seeking vaccination [42]. Likewise, individuals experiencing financial hardships during the pandemic exhibited lower vaccine uptake, supporting findings from previous studies [46,47]. Individuals facing financial insecurity may prioritize work over vaccination. The fear of income loss, particularly in the absence of social support such as paid leave, further exacerbates vaccine hesitancy [48]. 

Vaccine hesitancy was more pronounced among those who had no concern about illness for themselves or others. This finding aligns with parallel findings in existing literature, which suggests that hesitancy arises from a sense of complacency among individuals who do not perceive infection as a risk or who downplay the severity of the disease [20,49,50]. This perception of personal risk also influences adherence to other recommended safety measures for COVID-19, such as mask-wearing and social distancing [51]. Vaccine acceptance presents a more distinctly polarizing behavior, however, as the science and side effects are challenged by misinformation and concerns about long-term vaccine effects. Even individuals with first-hand experience, either in treating COVID-19 patients or having had the virus themselves, showed prolonged hesitancy. This finding emphasizes the fact that deeply rooted distrust can persist within many communities, and addressing this distrust and the associated misconceptions through appropriate public health messaging would be essential [52]. Additionally, for those who remain hesitant, it may be more effective to shift the focus from vaccination to individual safety as well as safety for their loved ones [53]. 

Our findings indicate that current smokers are more likely to be unvaccinated against COVID-19 than non-smokers. Bacerra et al. found that smokers were twice as likely as non-smokers to be unvaccinated [54]. In a study conducted by Moon et al., smokers were found to be 27% more likely to be unvaccinated than non-smokers [36,54]. This result is consistent with another finding in our survey, according to which individuals who are relatively unconcerned with illness themselves were more likely to remain unvaccinated. Additionally, our study suggests that individuals with a history of COVID-19 infection are more likely to be unvaccinated. Evidence from a study conducted by Huang et al. supports this idea, showing that individuals who have recovered from COVID-19 were more likely to be unvaccinated than individuals with no history of infection [55].

We found that individuals with diabetes, hypertension, or obesity were more likely to be vaccinated. However, empirical evidence regarding the presence of pre-existing conditions as factors influencing vaccine uptake varies. For instance, in a study conducted by Kizilkaya et al., obese patients were observed to be more likely to be unvaccinated [56]. Another study reported a higher prevalence of unvaccinated individuals among those with chronic conditions [57]. A meta-analysis by Bianchi et al. identified a high prevalence of unvaccinated status among individuals who are diabetic (28%) [58]. In Italy, research indicated a high prevalence of unvaccinated status among diabetic individuals exhibiting lower adherence to medications and lesser concerns about their health, suggesting health-related concerns as potential moderators of vaccine hesitancy among those with chronic diseases [59]. In contrast, a study conducted by Tsai et al. found that individuals with certain pre-existing conditions, such as cancer, were more inclined to receive the vaccine than those without cancer [60]. Perceived risk and infection severity may incentivize patients with immune-mediated diseases to seek out the COVID-19 vaccine [57,61].

This study has several limitations that warrant consideration. First, due to the cross-sectional nature of the study, temporality cannot be established. Second, we acknowledge the low response rate for our survey, which may potentially introduce sampling bias. Additionally, individuals willing to participate in the study may have differed significantly from those who chose not to participate. However, the alignment of our findings with national figures reported by organizations such as the CDC is reassuring regarding the representativeness of the sample. Third, because this was an interviewer-administered survey, it is susceptible to interviewer bias, posing a risk to validity. To mitigate this risk, we provided comprehensive training to all interviewers before beginning data collection. Fourth, differing exposure histories between respondents may have introduced recall bias into the study due to varying abilities to recollect events. Fifth, some questions regarding vaccine intentions were measured on a scale ranging from “not likely at all” to “extremely likely,” which may be affected by social desirability bias. Efforts were made during the interviewer training to present questions in a neutral, non-judgmental manner. Despite these limitations, this study can provide assurance through certain strengths. The larger sample obtained across Texas through phone interviews enhances the generalizability of the results to the entire state. Additionally, the phone surveys were administered both in English and Spanish, expanding the inclusivity of the study and enhancing the breadth of representation of the Texas population, which consists of over 40% Hispanics.

## 5. Conclusions

This study provided a wealth of data pertaining to vaccine uptake in Texas. We found that hesitation to take the COVID-19 vaccine was influenced by various contextual factors that shaped individuals’ daily lives. Our study examined the effects of race, ethnicity, and other individual characteristics on vaccination decisions statewide, informing public health officials and providers with valuable insights into factors that affect how individuals across a wide range of demographic and other population markers view the availability of vaccines as a strategy for avoiding COVID-19 infection or serious illness. Effecting change requires recognizing personal experiences and addressing underlying distrust that contributes to reluctance to learn new information. Strategies for promoting positive attitudes and behaviors towards the COVID-19 vaccine should be tailored for specific communities, considering factors such as race, education, religious beliefs, and geography. It is also important to acknowledge personal rights and political ideologies in vaccination efforts. Public health interventions designed to address vaccine hesitancy must take multiple complex factors into consideration at the community level, as there is no one-size-fits-all approach. 

## Figures and Tables

**Table 1:** Baseline Characteristics for Community Respondents by Vaccination Status (Unweighted).

Respondents, N (%) (Unless Otherwise Indicated)				
	Overall	Unvaccinated	Vaccinated	*p*-Value *
**Total Number of Respondents**	19,502	4297	15,205	
**PHR**				<0.001
**6/5S**	4913 (25.7)	993 (23.5)	3920 (26.3)	
**1**	456 (2.4)	152 (3.6)	304 (2.0)	
**11**	1224 (6.4)	182 (4.3)	1042 (7.0)	
**2/3**	5246 (27.4)	1224 (28.9)	4022 (27.0)	
**4/5N**	1521 (8.0)	503 (11.9)	1018 (6.8)	
**7**	2638 (13.8)	537 (12.7)	2101 (14.1)	
**8**	2144 (11.2)	421 (10.0)	1723 (11.6)	
**9/10**	983 (5.1)	219 (5.2)	764 (5.1)	
**Missing**	377	66	311	
**Age Group**				<0.001
**Age 18–39**	5299 (29.2)	1523 (38.5)	3776 (26.6)	
**Age 40–49**	3097 (17.1)	770 (19.5)	2327 (16.4)	
**Age 50–64**	5130 (28.3)	1111 (28.1)	4019 (28.3)	
**Age 65+**	4608 (25.4)	547 (13.8)	4061 (28.6)	
**Missing**	1368	346	1022	
**Sex/Gender**				<0.001
**Female**	9945 (51.2)	1889 (44.1)	8056 (53.1)	
**Male**	9213 (47.4)	2315 (54.1)	6898 (45.5)	
**Other**	283 (1.5)	78 (1.8)	205 (1.4)	
**Missing**	61	15	46	
**Race/Ethnicity**				<0.001
**White**	9690 (50.7)	2378 (57.2)	7312 (48.9)	
**Asian**	574 (3.0)	31 (0.7)	543 (3.6)	
**Black**	1983 (10.4)	321 (7.7)	1662 (11.1)	
**Hispanic**	5619 (29.4)	1033 (24.9)	4586 (30.6)	
**Multiracial**	463 (2.4)	139 (3.3)	324 (2.2)	
**Other**	792 (4.1)	252 (6.1)	540 (3.6)	
**Missing**	381	143	238	
**Language**				<0.001
**English**	16,763 (86.1)	3862 (90.1)	12,901 (85.0)	
**Other**	537 (2.8)	88 (2.1)	449 (3.0)	
**Spanish**	2160 (11.1)	337 (7.9)	1823 (12.0)	
**Missing**	42	10	32	
**Work**	12,286 (63.3)	3012 (70.6)	9274 (61.3)	<0.001
**Missing**	103	29	74	
**Work Situation**				<0.001
**Remote**	1591 (13.1)	304 (10.2)	1287 (14.0)	
**In-person**	7199 (59.2)	1932 (64.9)	5267 (57.3)	
**Hybrid**	3380 (27.8)	743 (24.9)	2637 (28.7)	
**Missing**	7332	1318	6014	
**No Work Reason**				<0.001
**Temporary lay-off**	176 (2.5)	37 (3.0)	139 (2.4)	
**Voluntary separation**	144 (2.0)	37 (3.0)	107 (1.8)	
**Permanently laid off**	137 (1.9)	31 (2.5)	106 (1.8)	
**Retired**	4184 (59.4)	525 (42.4)	3659 (63.0)	
**Student**	470 (6.7)	100 (8.1)	370 (6.4)	
**Other**	1936 (27.5)	508 (41.0)	1428 (24.6)	
**Missing**	12,455	3059	9396	
**No Work COVID Related**	91 (28.7)	21 (29.6)	70 (28.5)	0.97
**Missing**	19,185	4226	14,959	
**Insurance §**				<0.001
**Other**	1280 (6.9)	336 (8.4)	944 (6.5)	
**Private (+)**	10,311 (55.8)	2133 (53.4)	8178 (56.4)	
**Public**	4996 (27.0)	881 (22.1)	4115 (28.4)	
**Uninsured**	1908 (10.3)	645 (16.1)	1263 (8.7)	
**Missing**	1007	302	705	
**Cancer**	895 (4.6)	141 (3.3)	754 (5.0)	<0.001
**Missing**	5	3	2	
**Chronic Disease**	973 (5.0)	153 (3.6)	820 (5.4)	<0.001
**Missing**	5	3	2	
**COPD**	565 (2.9)	109 (2.5)	456 (3.0)	0.12
**Missing**	5	3	2	
**Heart Disease**	1447 (7.4)	238 (5.5)	1209 (8.0)	<0.001
**Missing**	5	3	2	
**Obesity**	2623 (13.5)	428 (10.0)	2195 (14.4)	<0.001
**Missing**	5	3	2	
**Sickle Cell**	114 (0.6)	27 (0.6)	87 (0.6)	0.75
**Missing**	5	3	2	
**Diabetes**	2424 (12.4)	329 (7.7)	2095 (13.8)	<0.001
**Missing**	5	3	2	
**Immunocompromised**	217 (1.1)	35 (0.8)	182 (1.2)	0.04
**Missing**	*5*	*3*	*2*	
**Smoker**	1719 (8.8)	533 (12.4)	1186 (7.8)	<0.001
**Missing**	*5*	*3*	*2*	
**Tuberculosis**	100 (0.5)	29 (0.7)	71 (0.5)	0.12
**Missing**	5	3	2	
**HIV**	78 (0.4)	27 (0.6)	51 (0.3)	0.01
**Missing**	5	3	2	
**Hypertension**	3370 (17.3)	438 (10.2)	2932 (19.3)	<0.001
**Missing**	18	5	13	
**Other Disease**	1548 (8.2)	279 (6.8)	1269 (8.7)	<0.001
**Missing**	725	172	553	
**Concern for Illness: Personal**				<0.001
**Not concerned at all**	8143 (42.0)	3094 (72.3)	5049 (33.4)	
**Somewhat not concerned**	2465 (12.7)	368 (8.6)	2097 (13.9)	
**Neither**	305 (1.6)	75 (1.8)	230 (1.5)	
**Somewhat concerned**	6197 (31.9)	584 (13.7)	5613 (37.1)	
**Extremely concerned**	2295 (11.8)	157 (3.7)	2138 (14.1)	
**Missing**	97	19	78	
**Concern for Illness: Others**				<0.001
**Not concerned at all**	6224 (32.1)	2352 (55.2)	3872 (25.6)	
**Somewhat not concerned**	1814 (9.4)	386 (9.1)	1428 (9.4)	
**Neither**	258 (1.3)	84 (2.0)	174 (1.2)	
**Somewhat concerned**	6562 (33.8)	1029 (24.2)	5533 (36.6)	
**Extremely concerned**	4530 (23.4)	407 (9.6)	4123 (27.3)	
**Missing**	114	39	75	
**COVID History**				<0.001
**No**	9976 (52.6)	1719 (41.9)	8257 (55.6)	
**Yes, medical care**	4055 (21.4)	967 (23.6)	3088 (20.8)	
**Yes, no medical care**	4934 (26.0)	1419 (34.6)	3515 (23.7)	
**Missing**	537	192	345	
**Struggle to Pay**	4039 (20.8)	1108 (25.9)	2931 (19.3)	<0.001
**Missing**	69	21	48	

N = number of respondents; PHR = Public Health Region. * *p*-values represent the significance level of the chi-square test of independence between randomized groups for binary and categorical variables or the one-way analysis of variance (ANOVA) between randomized groups for continuous variables. § Insurance category of Private (+) includes all private insurance options; Public includes Medicare, Medicaid, Medigap, CHIP, Military, Indian, State-Sponsored, and Affordable Care Act options; and Other includes those who have insurance but do not know which option and those with options not listed. In the comparison of sex groups, “missing” was excluded. Note that questions with higher missing counts were only asked if appropriate, so not every respondent had the option to answer. For instance, only respondents who claimed not to work answered “No Work Reason” and “No Work COVID Related.”

**Table 2:** Adjusted ‡ Odds Ratios for Being Unvaccinated Among Community Respondents.

	With All Variables			With LASSO Selected Variables		
	Odds Ratio	95% CI	*p*-Value *	Odds Ratio	95% CI	*p*-Value *
**PHR**			<0.001			<0.001
**6/5S**	-	-		-	-	
**1**	2.00	1.57, 2.54		2.00	1.57, 2.54	
**11**	0.72	0.60, 0.87		0.72	0.60, 0.87	
**2/3**	1.14	1.01, 1.28		1.14	1.01, 1.28	
**4/5N**	1.88	1.53, 2.31		1.88	1.53, 2.31	
**7**	0.94	0.80, 1.09		0.94	0.80, 1.09	
**8**	0.94	0.80, 1.10		0.94	0.80, 1.10	
**9/10**	1.03	0.84, 1.25		1.03	0.84, 1.25	
**Age Group**			<0.001			<0.001
**Age 18–39**	-	-		-	-	
**Age 40–49**	0.78	0.69, 0.88		0.78	0.69, 0.88	
**Age 50–64**	0.58	0.51, 0.65		0.58	0.51, 0.65	
**Age 65+**	0.28	0.23, 0.34		0.28	0.23, 0.34	
**Sex/Gender**			0.03			0.03
**Female**	-	-		-	-	
**Male**	1.11	1.01, 1.21		1.11	1.01, 1.21	
**Race/Ethnicity**			<0.001			<0.001
**White**	-	-		-	-	
**Asian**	0.14	0.10, 0.19		0.14	0.10, 0.19	
**Black**	0.71	0.61, 0.83		0.71	0.61, 0.83	
**Hispanic**	0.73	0.65, 0.81		0.73	0.65, 0.81	
**Multiracial**	1.10	0.84, 1.43		1.10	0.84, 1.43	
**Other**	1.29	0.75, 2.20		1.29	0.75, 2.20	
**Language**			<0.001			<0.001
**English**	-	-		-	-	
**Other**	0.59	0.42, 0.81		0.59	0.42, 0.81	
**Spanish**	0.59	0.51, 0.69		0.59	0.51, 0.69	
**Work**	0.92	0.83, 1.03	0.17	0.92	0.83, 1.03	0.17
**Insurance §**			<0.001			<0.001
**Other**	-	-		-	-	
**Private (+)**	0.76	0.65, 0.90		0.76	0.65, 0.90	
**Public**	0.87	0.72, 1.06		0.87	0.72, 1.06	
**Uninsured**	1.38	1.14, 1.67		1.38	1.14, 1.67	
**Cancer**	0.96	0.73, 1.26	0.79	0.96	0.73, 1.26	0.79
**Chronic Disease**	0.95	0.72, 1.24	0.72	0.95	0.72, 1.24	0.72
**COPD**	1.21	0.83, 1.73	0.32	1.21	0.83, 1.73	0.32
**Heart Disease**	1.22	0.97, 1.52	0.10	1.22	0.97, 1.52	0.10
**Obesity**	0.69	0.59, 0.81	<0.001	0.69	0.59, 0.81	<0.001
**Sickle Cell**	0.69	0.32, 1.34	0.28	0.69	0.32, 1.34	0.28
**Diabetes**	0.75	0.63, 0.90	0.002	0.75	0.63, 0.90	0.002
**Immunocompromised**	0.76	0.42, 1.30	0.33	0.76	0.42, 1.30	0.33
**Smoker**	1.56	1.36, 1.80	<0.001	1.56	1.36, 1.80	<0.001
**Tuberculosis**	1.79	0.95, 3.27	0.07	1.79	0.95, 3.27	0.07
**HIV**	0.82	0.34, 1.84	0.65	0.82	0.34, 1.84	0.65
**Hypertension**	0.74	0.63, 0.86	<0.001	0.74	0.63, 0.86	<0.001
**Other Disease**	1.10	0.93, 1.31	0.27	1.10	0.93, 1.31	0.27
**Concern for Illness: Personal**	0.38	0.34, 0.42	<0.001	0.38	0.34, 0.42	<0.001
**Concern for Illness: Others**	0.39	0.35, 0.43	<0.001	0.39	0.35, 0.43	<0.001
**COVID History**			<0.001			<0.001
**No**	-	-		-	-	
**Yes, medical care**	1.47	1.31, 1.65		1.47	1.31, 1.65	
**Yes, no medical care**	1.38	1.24, 1.52		1.38	1.24, 1.52	
**Struggle to Pay**	1.62	1.46, 1.79	<0.001	1.62	1.46, 1.79	<0.001

CI = Confidence Interval; PHR = Public Health Region. ‡ Adjusted for each covariate shown, in addition to all others presented in the table. * *p*-values represent the significance level of the likelihood ratio test for each factor within the model. § Insurance category of Private (+) includes all private insurance options; Public includes Medicare, Medicaid, Medigap, CHIP, Military, Indian, State-Sponsored, and Affordable Care Act options; and Other includes those who have insurance but do not know which option and those with options not listed. We excluded the “Other” category from the sex variable in the adjusted analysis since we did not have population estimates to assist with weighting this category. Note that all variables were selected during LASSO regression. Also, note that weighted data were used in the multiple logistic regression models represented above.

**Table 3:** Adjusted ‡ Odds Ratios for Being Likely to Receive the Vaccine in the Next 3 Months Among Unvaccinated Community Respondents, using LASSO Variable Selection.

	Odds Ratio	95% CI	*p*-Value *
**PHR**			0.41
**6/5S**	-	-	
**1**	2.60	1.20, 5.37	
**11**	1.41	0.66, 2.88	
**2/3**	1.21	0.76, 1.94	
**4/5N**	1.00	0.50, 1.90	
**7**	1.42	0.79, 2.52	
**8**	1.14	0.60, 2.09	
**9/10**	1.46	0.68, 2.94	
**Age Group**			<0.001
**Age 18–39**	-	-	
**Age 40–49**	0.53	0.33, 0.84	
**Age 50–64**	0.37	0.21, 0.61	
**Age 65+**	1.06	0.60, 1.85	
**Race/Ethnicity**			0.01
**White**	-	-	
**Asian**	0.82	0.04, 4.78	
**Black**	2.04	1.14, 3.55	
**Hispanic**	2.10	1.33, 3.29	
**Multiracial**	1.03	0.34, 2.53	
**Other**	0.82	0.28, 1.95	
**Language**			<0.001
**English**	-	-	
**Other**	1.47	0.40, 4.23	
**Spanish**	2.73	1.67, 4.46	
**Work**	0.59	0.40, 0.85	0.006
**Insurance §**			0.24
**Other**	-	-	
**Private (+)**	0.86	0.47, 1.64	
**Public**	0.70	0.36, 1.41	
**Uninsured**	1.18	0.63, 2.33	
**COPD**	1.92	0.72, 4.49	0.18
**Diabetes**	0.41	0.17, 0.85	0.01
**Concern for Illness: Personal**	3.35	2.31, 4.87	<0.001
**Concern for Illness: Others**	1.98	1.37, 2.88	<0.001
**Struggle to Pay**	1.32	0.93, 1.87	0.12

CI = Confidence Interval; PHR = Public Health Region. ‡ Adjusted for each covariate shown, in addition to all others presented in the table. * *p*-values represent the significance level of the likelihood ratio test for each factor within the model. § Insurance category of Private (+) includes all private insurance options; Public includes Medicare, Medicaid, Medigap, CHIP, Military, Indian, State-Sponsored, and Affordable Care Act options; and Other includes those who have insurance but do not know which option and those with options not listed. Note that unweighted data were used to fit the multiple logistic regression model represented above.

**Table 4:** Baseline Characteristics for Community Respondents with Unknown or Unvaccinated Status by Barrier Experience.

Respondents, N (%) (Unless Otherwise Indicated)				
	Overall	No Barriers	Barriers	*p*-Value *
**Total Number of Respondents**	4313	4202	111	
**PHR**				0.78
**6/5S**	996 (23.5)	974 (23.5)	22 (20.0)	
**1**	154 (3.6)	149 (3.6)	5 (4.5)	
**11**	184 (4.3)	176 (4.3)	8 (7.3)	
**2/3**	1239 (29.2)	1204 (29.1)	35 (31.8)	
**4/5N**	496 (11.7)	484 (11.7)	12 (10.9)	
**7**	537 (12.6)	523 (12.6)	14 (12.7)	
**8**	420 (9.9)	410 (9.9)	10 (9.1)	
**9/10**	221 (5.2)	217 (5.2)	4 (3.6)	
**Missing**	66	65	1	
**Age Group**				0.22
**Age 18–39**	1528 (38.7)	1491 (38.7)	37 (39.8)	
**Age 40–49**	767 (19.4)	753 (19.5)	14 (15.1)	
**Age 50–64**	1111 (28.1)	1088 (28.2)	23 (24.7)	
**Age 65+**	543 (13.8)	524 (13.6)	19 (20.4)	
**Missing**	364	346	18	
**Sex/Gender**				0.72
**Female**	1913 (44.5)	1862 (44.5)	51 (45.9)	
**Male**	2307 (53.7)	2250 (53.7)	57 (51.4)	
**Other**	78 (1.8)	75 (1.8)	3 (2.7)	
**Missing**	15	15	0	
**Race/Ethnicity**				0.001
**White**	2377 (57.0)	2333 (57.4)	44 (41.9)	
**Asian**	29 (0.7)	26 (0.6)	3 (2.9)	
**Black**	324 (7.8)	317 (7.8)	7 (6.7)	
**Hispanic**	1037 (24.9)	1000 (24.6)	37 (35.2)	
**Multiracial**	144 (3.5)	137 (3.4)	7 (6.7)	
**Other**	256 (6.1)	249 (6.1)	7 (6.7)	
**Missing**	146	140	6	
**Language**				<0.001
**English**	3876 (90.1)	3791 (90.5)	85 (76.6)	
**Other**	89 (2.1)	83 (2.0)	6 (5.4)	
**Spanish**	337 (7.8)	317 (7.6)	20 (18.0)	
**Missing**	11	11	0	
**Work**	3026 (70.7)	2963 (71.0)	63 (57.3)	0.003
**Missing**	30	29	1	
**Work Situation**				0.56
**Remote**	303 (10.1)	299 (10.2)	4 (6.5)	
**In-person**	1927 (64.4)	1887 (64.4)	40 (64.5)	
**Hybrid**	761 (25.4)	743 (25.4)	18 (29.0)	
**Missing**	1322	1273	49	
**No Work Reason**				0.10
**Temporary lay-off**	36 (2.9)	35 (2.9)	1 (2.2)	
**Voluntary separation**	38 (3.1)	38 (3.2)	0 (0.0)	
**Permanently laid off**	30 (2.4)	27 (2.3)	3 (6.5)	
**Retired**	521 (42.2)	508 (42.7)	13 (28.3)	
**Student**	100 (8.1)	94 (7.9)	6 (13.0)	
**Other**	511 (41.3)	488 (41.0)	23 (50.0)	
**Missing**	3077	3012	65	
**No Work COVID Related**	20 (28.2)	19 (27.1)	1 (100.0)	0.63
**Missing**	4242	4132	110	
**Insurance §**				0.001
**Other**	338 (8.4)	330 (8.5)	8 (7.8)	
**Private (+)**	2150 (53.7)	2113 (54.2)	37 (35.9)	
**Public**	876 (21.9)	841 (21.6)	35 (34.0)	
**Uninsured**	641 (16.0)	618 (15.8)	23 (22.3)	
**Missing**	308	300	8	
**Cancer**	142 (3.3)	133 (3.2)	9 (8.1)	0.009
**Missing**	3	3	0	
**Chronic Disease**	151 (3.5)	135 (3.2)	16 (14.4)	<0.001
**Missing**	3	3	0	
**COPD**	109 (2.5)	101 (2.4)	8 (7.2)	0.004
**Missing**	3	3	0	
**Heart Disease**	236 (5.5)	221 (5.3)	15 (13.5)	<0.001
**Missing**	3	3	0	
**Obesity**	426 (9.9)	410 (9.8)	16 (14.4)	0.15
**Missing**	3	3	0	
**Sickle Cell**	29 (0.7)	21 (0.5)	8 (7.2)	<0.001
**Missing**	3	3	0	
**Diabetes**	325 (7.5)	311 (7.4)	14 (12.6)	0.06
**Missing**	3	3	0	
**Immunocompromised**	36 (0.8)	32 (0.8)	4 (3.6)	0.007
**Missing**	3	3	0	
**Smoker**	530 (12.3)	514 (12.2)	16 (14.4)	0.59
**Missing**	3	3	0	
**Tuberculosis**	30 (0.7)	27 (0.6)	3 (2.7)	0.046
**Missing**	3	3	0	
**HIV**	26 (0.6)	24 (0.6)	2 (1.8)	0.30
**Missing**	3	3	0	
**Hypertension**	436 (10.1)	422 (10.1)	14 (12.6)	0.47
**Missing**	5	5	0	
**Other Disease**	282 (6.8)	263 (6.5)	19 (18.1)	<0.001
**Missing**	171	165	6	
**Concern for Illness: Personal**				<0.001
**Not concerned at all**	3104 (72.3)	3060 (73.1)	44 (40.0)	
**Somewhat not concerned**	375 (8.7)	359 (8.6)	16 (14.5)	
**Neither**	76 (1.8)	72 (1.7)	4 (3.6)	
**Somewhat concerned**	588 (13.7)	563 (13.4)	25 (22.7)	
**Extremely concerned**	153 (3.6)	132 (3.2)	21 (19.1)	
**Missing**	17	16	1	
**Concern for Illness: Others**				<0.001
**Not concerned at all**	2347 (54.9)	2313 (55.5)	34 (31.2)	
**Somewhat not concerned**	393 (9.2)	383 (9.2)	10 (9.2)	
**Neither**	89 (2.1)	83 (2.0)	6 (5.5)	
**Somewhat concerned**	1046 (24.5)	1012 (24.3)	34 (31.2)	
**Extremely concerned**	401 (9.4)	376 (9.0)	25 (22.9)	
**Missing**	37	35	2	
**COVID History**				0.19
**No**	1734 (42.2)	1692 (42.2)	42 (41.2)	
**Yes, medical care**	958 (23.3)	927 (23.1)	31 (30.4)	
**Yes, no medical care**	1416 (34.5)	1387 (34.6)	29 (28.4)	
**Missing**	205	196	9	
**Struggle to Pay**	1103 (25.7)	1050 (25.1)	53 (48.2)	<0.001
**Missing**	23	22	1	

N = number of respondents; PHR = Public Health Region. * *p*-values represent the significance level of the chi-square test of independence between randomized groups for binary and categorical variables or the one-way analysis of variance (ANOVA) between randomized groups for continuous variables. § Insurance category of Private (+) includes all private insurance options; Public includes Medicare, Medicaid, Medigap, CHIP, Military, Indian, State-Sponsored, and Affordable Care Act options; and Other includes those who have insurance but do not know which option and those with options not listed. Note that questions with higher missing counts were only asked if appropriate, so not every respondent had the option to answer. For instance, only respondents who claimed not to work answered “No Work Reason” and “No Work COVID Related.”

## Data Availability

Restrictions apply to the datasets. The researchers have a contractual agreement with the owner of the data that restricts data sharing with third parties.
